# Prognostic Impact of APOBEC3B Expression in Metastatic Urothelial Carcinoma and Its Association with Tumor-Infiltrating Cytotoxic T Cells

**DOI:** 10.3390/curroncol28030154

**Published:** 2021-04-28

**Authors:** Hyunho Kim, Okran Kim, Myung Ah Lee, Ji Youl Lee, Sung-Hoo Hong, U-Syn Ha, Kwangil Yim, In-Ho Kim

**Affiliations:** 1 Division of Medical Oncology, Department of Internal Medicine, St. Vincent’s Hospital, The Catholic University of Korea, Suwon 16247, Korea; h2kim@catholic.ac.kr; 2 Cancer Research Institute, College of Medicine, The Catholic University of Korea, Seoul 06591, Korea; loveju@catholic.ac.kr; 3 Division of Medical Oncology, Department of Internal Medicine, Seoul St. Mary’s Hospital, The Catholic University of Korea, Seoul 06591, Korea; angelamd@catholic.ac.kr; 4 Department of Urology Cancer Center, Seoul St. Mary’s Hospital, The Catholic University of Korea, Seoul 06591, Korea; uroljy@catholic.ac.kr (J.Y.L.); toomey@catholic.ac.kr (S.-H.H.); ushamd@catholic.ac.kr (U.-S.H.); 5 Department of Hospital Pathology, Uijeongbu St. Mary’s Hospital, The Catholic University of Korea, Uijeongbu 11765, Korea

**Keywords:** mutagen, tumor-infiltrating T-lymphocyte, cytotoxic, urologic neoplasm, prognosis

## Abstract

APOBEC3B enzymes are endogenous carcinogenic mutagens. Metastatic urothelial carcinomas often harbor APOBEC3B-mediated mutations in which tCw to T or G substitution occurs. Here, we evaluated patient survival and CD8+ T-cell density according to APOBEC3B expression in patients with metastatic urothelial carcinoma who underwent cytotoxic chemotherapy. We performed a retrospective study on 94 patients with urothelial carcinoma who were treated with first line palliative chemotherapy. APOBEC3B expression and CD8+/CD3+ ratio of tumor-infiltrating lymphocytes were evaluated using immunohistochemistry. Kaplan–Meier survival curves were generated and the log-rank test was employed. The association between APOBEC3B expression and tumor-infiltrating lymphocytes was analyzed using Pearson’s chi-squared test. High APOBEC3B expression was detected in 71 of the 94 patients (75.5%). The median overall survival was longer in patients with high APOBEC3B expression (15 months) than in those with low expression (*p* = 0.045). The hazard ratio obtained based on the Cox regression analysis was 0.292 (95% confidence interval 0.118–0.723, *p* = 0.008). APOBEC3B expression was associated with the CD8+/CD3+ ratio (2.914, 95% confidence interval 1.030–8.249, *p* = 0.039). Collectively, APOBEC3B expression was an independent prognostic factor in patients with metastatic urothelial carcinoma treated with platinum-based chemotherapy. Tumor-infiltrating cytotoxic T cells were associated with APOBEC3B expression.

## 1. Introduction

Urothelial carcinoma is a histopathological subtype malignancy that originates from the urinary tract epithelial linings from the renal calyces to the urethra [1]. The most common primary site of urothelial carcinomas (90%–95%) is the bladder [1,2]. The response rate of metastatic bladder cancer to platinum-based systemic chemotherapy, the main treatment option, is high (at 40%–60%) compared with that of other solid cancers [3]. In addition, recently, immune checkpoint inhibitors (ICIs), as a second line or higher treatment for metastatic bladder cancer, have been shown to prolong patient survival [4]. However, the median overall survival (OS) is around 10–12 months, and prognosis remains poor [3,4,5]. Therefore, it is important to analyze the prognosis of metastatic urothelial carcinoma and to develop appropriate treatment strategies to improve patient survival.

The risk factors for bladder cancer are exposure to chemicals, such as those in tobacco, chronic irritation, and abnormalities in genes related to cell cycle regulation [6]. In terms of gene abnormalities, an analysis of the frequency of somatic mutations using 3083 tumor–normal paired specimens from 27 types of cancer revealed that bladder cancer has a mean non-synonymous mutation rate of 8.2 mutations/megabyte (MB) and a median rate of 5.8 mutations/MB [7,8]. This is the third highest frequency rate, following that of malignant melanoma and non-small cell lung cancer [7]. An analysis of the mutation spectrum showed that the major mutation pattern differs for each cancer type, such as TpC > mutation for bladder cancer, C > T for malignant melanoma, C > A for lung cancer, and CpG > T for gastrointestinal track cancer [7]. Furthermore, according to an analysis of somatic mutations in human cancers available in the Catalogue Of Somatic Mutations In Cancer (COSMIC) cancer database, bladder cancer harbors 1, 2, 5, 10, and 13 mutation signatures [9]. Of these, signatures 2 and 13 overlap with the aforementioned TpC > mutation pattern [7], which is a well-known form of apolipoprotein B mRNA editing enzyme (APOBEC)-mediated mutation in which the C in the tCw triplet motif is changed to a T or G [10,11].

APOBEC enzymes are a family of evolutionarily conserved cytidine deaminases and are endogenous mutagens that induce somatic driver and passenger gene mutations in human cancers through cytidine deaminase [11]. Currently, the most well-known epidemiologic cause of bladder cancer is smoking, which accounts for 50% of cases [6]. The mutational pattern C > A is known to be related to smoking and is often observed in lung cancer, whereas the tCw > T or G mutational pattern is mainly observed in bladder cancer [7,11]. This suggests that, in addition to smoking, APOBEC-mediated mutations are a major contributor to bladder cancer development. Therefore, it can be assumed that tumor biology and bladder cancer prognosis may differ according to the APOBEC-related mutagenesis involved. An analysis of a bladder cancer cohort in The Cancer Genome Atlas (TCGA) database revealed that the expression of APOBEC3B, which is one of several members of the APOBEC family, is upregulated and is associated with patient survival [12]. We hypothesized that these results may be associated with the infiltration of T lymphocytes into tumors as a result of an APOBEC-mediated high mutation burden. Therefore, in the present study, we investigated the prognosis of metastatic urothelial carcinoma according to APOBEC3B expression and analyzed tumor-infiltrating lymphocytes (TILs) by investigating tumor immunity. APOBEC3B expression was found to be an independent prognostic factor in patients with metastatic urothelial carcinoma, and tumor-infiltrating cytotoxic T cells were associated with APOBEC3B expression.

## 2. Results

### 2.1. Baseline Characteristics

Ninety-four patients were included in this retrospective study. From the total 94 patients analyzed, 80 showed disease progression and 80 died, as indicated in the medical records. The characteristics of these patients are presented in Table 1. Smoking history was not included as the relevant information was not available for most of the patients. A high expression of APOBEC3B was observed in 71 of the 94 patients (75.5%), similar to the TCGA bladder cancer cohort data for APOBEC-mediated mutation enrichment scores (83.5%) [12]. There were no significant differences according to the APOBEC3B expression status for treatment-related factors, namely, chemotherapy regimen, subsequent chemotherapy, or resection history (Table 1).

### 2.2. Prognostic Value of APOBEC3B for Patient Survival

**Figure 1:**
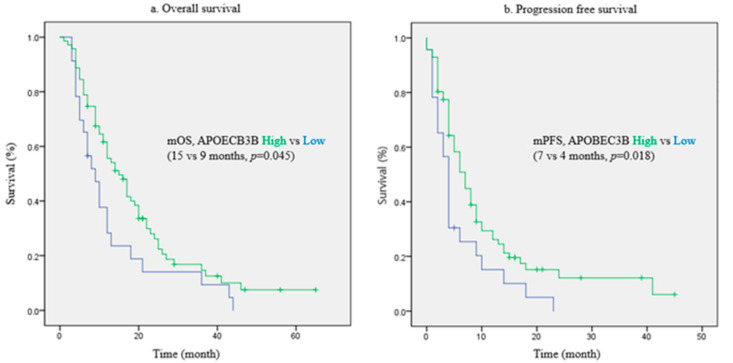
Survival curves according to APOBEC3B expression. (**a**) Overall survival, (**b**) Progression free survival.

The survival of patients was assessed according to the APOBEC3B expression status. The median OS was longer in patients with high APOBEC3B expression at 15 months than in those with low expression at nine months (*p* = 0.045 by log-rank test; Figure 1a). Similarly, the median progression-free survival (PFS) was longer in patients with high APOBEC3B expression at seven months than in those with low expression at four months (*p* = 0.018 by log-rank test; Figure 1b). We also analyzed the hazard ratio (HR) according to the APOBEC3B expression status using the Cox regression models. The multivariate analysis was adjusted for chemotherapy response, subsequent chemotherapy, surgery, Eastern Cooperative Oncology Group Performance Status (ECOG-PS), and chemotherapy regimen. These variables were found to be significant factors based on the log-rank test (Supplementary Table S1), and chemotherapy regimen is historically a significant factor [5]. The results presented in Table 2 showed that the APOBEC3B expression status was an independent prognostic factor for OS [HR 0.292, 95% confidence interval (CI) 0.118–0.723, *p* = 0.008], and it was also significant for PFS according to the Cox regression analysis (HR 0.335, 95% CI 0.139–0.806, *p* = 0.015).

### 2.3. Tumor-Infiltrating Lymphocytes (TIL) Differences According to the APOBEC3B Expression Status

As APOBEC3B is an endogenous carcinogenic mutagen by APOBEC-mediated cytidine deamination [10,11], we hypothesized that APOBEC mutation would result in the production of a tumor neoantigen and increased infiltration of T lymphocytes into intra- or peri-tumor tissues. This was evaluated by comparing CD8+/CD3+ TIL ratio between patients with high and low APOBEC3B expression. The results showed that low APOBEC3B expression was associated with a low CD8+/CD3+ TIL ratio (odds ratio 2.914, 95% CI 1.030–8.249, *p* = 0.039; Table 3). Interestingly, similar to APOBEC3B, there was a difference in the OS and PFS according to the CD8+/CD3+ TIL ratio (Supplementary Figure S1). These findings suggested that APOBEC3B expression associated with TILs in metastatic bladder cancer. With respect to tumor-infiltrating regulatory T cells, the FOXP3+/CD4+ TIL ratio was not associated with the APOBEC3B expression status (chi-squared test, *p* = 0.186; Supplementary Table S2), although a small number of patients (*n* = 10) presented a low FOXP3+/CD4+ TIL ratio among the 47 patients who showed high APOBEC3B expression with objective responses. The FOXP3+/CD4+ TIL ratio was not related to the CD8+/CD3+ TIL ratio (chi-squared test, *p* = 0.894).

### 2.4. Evaluation of APOBEC3B Expression as a Predictive Marker for Cytotoxic Chemotherapy

In a study of neoadjuvant chemotherapy for muscle invasive bladder cancer, TILs were found to be associated with a cisplatin-based chemotherapy response [13]. Therefore, considering the association of APOBEC3B expression with TILs, we expected that high APOBEC3B expression might be associated with a better cytotoxic effect for metastatic urothelial carcinoma. However, there was no significant difference in chemotherapy response according to the APOBEC3B status (*p* = 0.227; Table 4) and TILs (*p* = 0.559; Supplementary Table S3).

## 3. Discussion

In this study, we evaluated the prognostic significance of APOBEC3B expression in metastatic bladder cancer. In a previous comparative study of 14 cancer types, APOBEC-mediated mutations in bladder cancer were observed at a higher rate than those in other cancers [11]. APOBEC-mediated mutation has been reported to correlate with *APOBEC* mRNA expression, and APOBEC1, APOBEC3A, APOBEC3B, APOBEC3F, and APOBEC3G, among the APOBEC family members, have been statistically confirmed to correlate with mRNA expression [8]. APOBEC3B presented the strongest correlation (Spearman = 0.30, *p* < 0.001), and the expression level of APOBEC3B mRNA in bladder cancer was significantly higher than the median value of all samples of 14 cancer types evaluated in a previous study [11]. In addition, only APOBEC3B of the APOBEC family showed a significantly higher mRNA expression in bladder cancer than in normal tissue (*p* < 0.001) [8]. Therefore, we selected APOBEC3B from several APOBEC enzymes as the subject of this study. The survival curves were clearly grouped depending on the APOBEC3B expression status. Previous studies support our current results, although there are no studies on APOBEC3B specifically [14,15].

APOBEC3 is one of the cytidine deaminases involved in C to U editing [11]. It was first discovered in relation to a restriction factor against viral infection and adaptive immunity through antibody diversification [10,11]. Subsequently, APOBEC mutation of the tCw motif was identified, and it was found to be highly specific in single-stranded DNA and responsible for somatic mutations that may induce carcinogenesis and cancer evolution in various types of human solid cancers [10,11]. Gene mutations in bladder cancer resulting in a high expression of APOBEC are frequently observed in genes related to DNA damage response, chromatin modification, phosphatidylinositol-4,5-bisphosphate 3-kinase, catalytic subunit alpha (*PIK3CA*), and tumor protein p53 (*TP53*) [12]. In contrast, specimens with a low expression of APOBEC are reported to have mutations in *KRAS* and fibroblast growth factor receptor 3 (*FGFR3*) [12]. These distinct mutational characteristics suggest that the tumor biology of bladder cancer may be different between those with high and low expression of APOBEC3B and that the APOBEC3B activity may be associated with DNA damage response. A previous study in a breast cancer cell line reported that the loss of tumor suppressor genes, such as *TP53*, and DNA replication stress induced increased transcription of APOBEC3B [16]. Replication stress increases replication fork stalling, and exposure of single-stranded DNA may favor APOBEC3B function [11,17]. In addition, in APOBEC3A-expressing cells, inhibition of ataxia telangiectasia and Rad3-related protein (ATR), a protein involved in DNA damage response, results in an increase in apurinic/apyrimidinic (abasic) sites and the accumulation of single-stranded DNA, which drive cells into a replication catastrophe, including massive DNA breakage and cell death [18]. Therefore, we thought that increased APOBEC3B activity may accelerate the production of various neoantigens in bladder cancer cells. The difference in antigenicity may ultimately result in tumor immunity in APOBEC3B-expressing bladder cancer. However, the association between tumor mutation burden and TILs, which can reflect tumor immunity, is still unclear, and further study is needed in this regard [19].

We assessed intra-tumoral and peri-tumoral TILs, but not tumor mutation burden, in relation to APOBEC3B expression. A previous study reported that a high density of intra-tumoral CD8+ T cells is associated with longer OS (*p* = 0.02) [20]. Consistent with this finding, intra-tumoral and stromal TILs in breast cancer influence treatment outcomes [21]. In an analysis of TCGA RNA-sequencing data, the expression of the APOBEC3 family members correlated with T-cell markers in various types of solid cancers and APOBEC3G exhibited a strong correlation with activated cytotoxic T-cell markers, such as granzyme B (*GZMB*) and perforin (*PFR1*) in high-grade ovarian cancer (*r* = 0.6591, *p* < 0.0001 and *r* = 0.6422, *p* < 0.0001, respectively) [22]. With respect to tumor mutation burden, APOBEC3B expression is strongly correlated with the total mutation burden in bladder cancer (*r* = 0.3308, *p* < 0.001) [12]. These previous study results suggest that, in metastatic bladder cancer, the differences in survival curves depending on APOBEC3B expression may be associated with APOBEC-mediated mutations and TILs. Consistent with this, our study showed that there were distinct high-expressing and low-expressing APOBEC3B groups in terms of TILs and survival curves.

Our study results suggested that APOBEC3B was an independent prognostic factor for metastatic urothelial carcinoma and appeared to be associated with TILs, which can represent tumor immunity. However, there were limitations to our study. First, the immune response against cancer cells is substantially complicated and cannot be simply explained by only infiltrating cytotoxic T cells [10]. Many factors associated with immune suppressive activity are present in the tumor microenvironment, such as TGFβ, and tumor cells continue to evolve through mechanisms such as epithelial–mesenchymal transition, angiogenesis, and immune inhibitory molecule production, which help in promoting immune evasion [10]. We did not evaluate these various factors of the tumor microenvironment in the present study. Second, there is considerable evidence from previous studies regarding correlations between total non-synonymous mutation of cancer-associated genes and mRNA expression of *APOBEC3B* [11,12]. However, these studies were limited in relation to the association of mRNA expression with protein expression, especially in metastatic urothelial cancer. Therefore, based on the previous mRNA studies, we assumed that the high expression of APOBEC3B protein may lead to cancer-associated mutagenesis, and the first quartile of the APOBEC3B Histo-score (H-score) was determined as a cut-off value because the proportion of APOBEC3B mRNA expression was approximately 80%. Finally, our study was a retrospective study and inevitably was limited in the ability to adjust for various confounding factors. Given the total number of patients and cut-off value, there were fewer patients in the low-expression group than in the high-expression group. Moreover, of the total 94 carcinoma cases, 44 originated from the upper urinary tract and not from the bladder. A future well-designed prospective study is required to overcome these limitations and to verify our findings.

There are some interesting questions that our study raises. Recognition of tumor antigen is important for infiltrating cytotoxic T cells to operate in the immune system [10], and thus immunogenic cell death is needed to expose antigens in identifiable states to appropriate immune cells [23]. In this sense, platinum agents are well-known to be involved in immunogenic cell death [23]. Perhaps, this is related to immune surveillance and is a factor leading to a better prognosis of patients with high APOBEC expression in bladder cancer undergoing cytotoxic chemotherapy than patients with low APOBEC expression [10]. On the contrary, chemotherapy could induce replication stress and higher APOBEC3B expression, which may ultimately lead to the development of an increase in clones during chemotherapy for cancers resistant to cytotoxic drugs [10]. Taken together, APOBEC3B may have lasting effects on the initial cytotoxic chemotherapy response to acquired resistance.

Another question is whether APOBEC3B expression influences the effect of ICIs. In bladder cancer, anti-PD1 or PD-L1 monoclonal antibodies are among the therapeutic choices for treating patients with metastatic urothelial carcinoma [4]. However, in the present study, we did not evaluate the PD-L1 status, which is often used as a predictive marker for anti-PD1 or PD-L1 antibodies. A previous study reported that APOBEC3B expression was marginally associated with PD-L1 expression on immune cells in metastatic urothelial carcinoma (*p* = 0.05) [15]. Another study suggested that APOBEC-induced mutagenesis affects immune evasion of cancer cells through HLA mutations in urothelial carcinoma [10]. Contrary to a good response expected due to increased tumor mutation burden, these results support the possibility of resistance to ICIs in metastatic urothelial carcinoma by harboring APOBEC3B-mediated mutations. Therefore, besides cytotoxic chemotherapy, further studies are needed to evaluate the influence of *APOBEC3B* mutation on ICIs in urothelial carcinoma.

## 4. Materials and Methods

### 4.1. Study Population and Design

This was a retrospective study approved by the Institutional Review Board of the Seoul St. Mary’s Hospital of the Catholic University of Korea (KC18SESI0437). All the patients provided written informed consent for participating in the study. The study was conducted in patients treated with first line palliative chemotherapy for metastatic urothelial carcinoma from 2009 to May 2018 at Seoul St. Mary’s Hospital. All the diagnoses were confirmed pathologically. The chemotherapy regimens included gemcitabine plus cisplatin or gemcitabine plus carboplatin for patients who were ineligible to receive cisplatin. To evaluate treatment responses, we reviewed the results of computed tomography imaging. Radiological changes were assessed using Response Evaluation Criteria in Solid Tumors version 1.1 [24]. Other clinical factors reviewed were age, sex, ECOG-PS, primary site of urothelial carcinoma, initial cancer stage at diagnosis, history of tumor resection, and previous or subsequent chemotherapy. All the patients were followed until data lock (13 October 2019) or death.

### 4.2. Immunohistochemistry (IHC)

**Figure 2:**
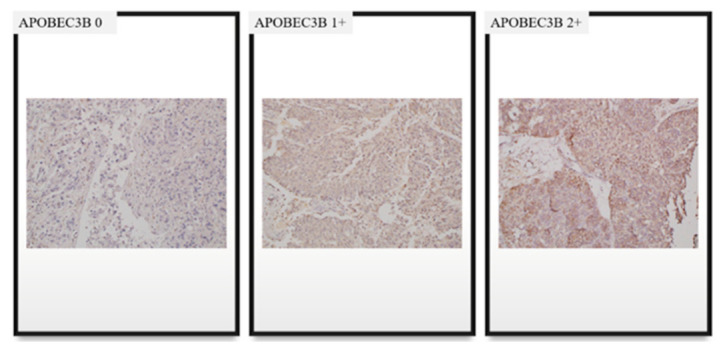
Immunohistochemistry staining of APOBEC3B.

**Figure 3:**
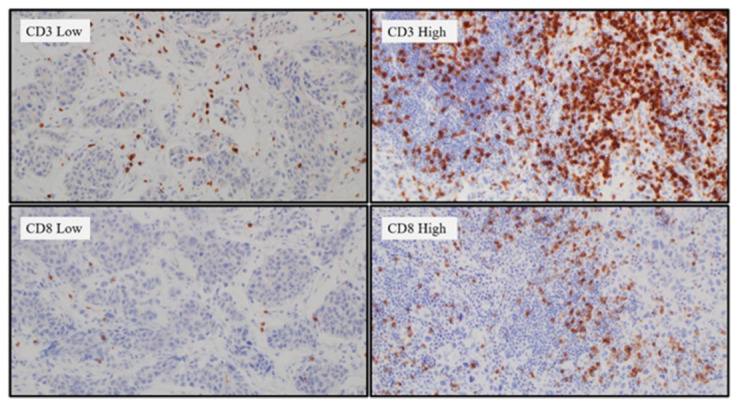
Immunohistochemistry staining of CD3+ and CD8+ T cells.

**Figure 4:**
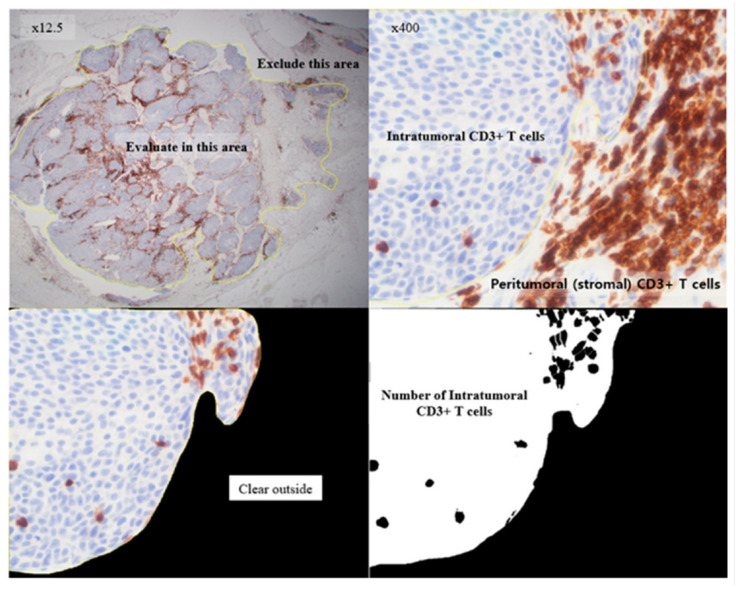
Counting of intratumoral CD3+ T cells using the ImageJ software.

A formalin-fixed, paraffin-embedded block containing both tumor and normal tissue was selected to serve as an internal control. The IHC analysis was performed as described previously [25]. The primary antibody against human APOBEC3B used in the procedure was a rabbit polyclonal anti-APOBEC3B antibody (Abcam, Cambridge, UK) at a concentration of 5 µg/mL. To analyze APOBEC3B expression, K.Y. (a pathologist) evaluated nucleus and cytoplasm staining of each sample using a semiquantitative score. The scores were on a scale of 0–300, and they were calculated by multiplying the staining intensity (0: no staining, 1: weak, 2: moderate, and 3: strong) with the percentage of cells (0–100%) at each intensity level (Figure 2). To analyze TILs, all cancer components on the entire slide were carefully evaluated and the most active foci were selected. Three areas were evaluated for CD3, CD4, CD8, and FOXP3 staining using 20× objective magnification. Two of the areas were focused on stromal TILs and one area focused on intra-tumoral TILs (Figure 3) [26]. The TILs were counted, and the area occupied by TILs per total stromal or tumoral area was calculated using the ImageJ software (National Institutes of Health, Bethesda, MD, USA) (Figure 4). The IHC staining was evaluated by a pathologist blinded to the patient outcomes.

### 4.3. Interpretation of APOBEC3B Expression and TILs Based on IHC Staining

To assess APOBEC3B expression, H-score was calculated in a range of 0 to 300 using the following formula: H-score = (3 × percentage of strongly staining) + (2 × percentage of moderately staining) + (percentage of weakly staining) [27]. The APOBEC3B enzymes are produced in the cytoplasm and transported to the nucleus to function as mutagens [11]. Therefore, we determined the H-scores for APOBEC3B expression in both cytoplasm and nucleus, and then added the values to interpret APOBEC3B expression. The patients were classified as high and low expressing depending on the combined nucleus and cytoplasm H-scores. The cut-off level of H-score was 90 for the first quartile. This refers to the fact that high APOBEC expression was reported to be approximately 80% in a previous study that analyzed the TCGA database [12].

To evaluate tumor-infiltrating cytotoxic and regulatory T cells, CD8+/CD3+ ratios and FOXP3+/CD4+ ratios, respectively, were calculated. Each ratio was calculated for stromal and intra-tumoral staining and summarized as a range of 0.00‒2.00. The patients were assigned low- and high-infiltrating groups depending on the values using a demarcation value of 1.00 for the two groups.

### 4.4. Statistical Analysis

The baseline characteristics of patients were compared using the Chi-squared test for each category. OS and PFS were calculated from the start date of gemcitabine plus platinum chemotherapy to the date of death or to the date of disease progression, respectively. Survival curves were generated using the Kaplan–Meier method and were compared using the log-rank test. Multivariate Cox regression models were used to verify the prognostic value of APOBEC3B. The correlation between APOBEC3B expression and TILs was analyzed using Pearson’s chi-squared test. Chemotherapy response was also analyzed using the Chi-squared test based on APOBEC3B expression and TILs. All statistical analyses were performed using the SPSS software (version 24; IBM Corp., Armonk, NY, USA). Results with a two-sided *p*-value of < 0.05 were considered statistically significant.

## 5. Conclusions

In summary, APOBEC3B expression was an independent prognostic factor for metastatic urothelial carcinoma treated with platinum-based chemotherapy. Tumor-infiltrating cytotoxic T cells associated with APOBEC3B expression. Therefore, the impact of APOBEC3B on the prognosis of metastatic urothelial carcinoma may involve increased T-cell immunity.

## Figures and Tables

**Table 1:** Baseline characteristics of the patients.

		Total (*n* = 94)	APOBEC3B Low * (*n* = 23)	APOBEC3B High ** (*n* = 71)	*p*-Value
Age	median (range)	68 (36–93)	68 (48–88)	70 (36–93)	
	<65	33	6	27	0.297
	≥65	61	17	44	
Sex	Female	22	5	17	0.828
	Male	72	18	54	
ECOG-PS ***	0	16	1	15	
	1	23	5	18	
	2	10	2	8	
	unknown	45	15	30	
Primary Site	Renal pelvis	22	8	14	0.314
	Ureter	22	4	18	
	Bladder	50	11	39	
Disease presentation	Recurrent	57	13	44	0.642
	Metastatic	37	10	27	
Metastatic site	Lymphnode only	36	10	26	0.556
	Liver, Lung, bone, others	58	13	45	
Regimen	Gem/Cis	29	4	25	0.108
	Gem/Carbo	65	19	46	
previous chemotherapy	No	83	21	62	1.000
	Yes	11	2	9	
Subsequent chemotherapy	No	43	9	34	0.464
	Yes	51	14	37	
Surgery	No	41	13	28	0.151
	Yes	53	10	43	
H-score of APOBEC3B	median (range)	110 (0–280)	70 (0–90)	120 (95–280)	

* Low: H-score ≤ 90, ** High, H-score > 90, H-score (nucleus + cytoplasm) = intensity × proportion. *** ECOG-PS (0, no performance restrictions; 1, fully ambulatory and able to carry out light work; 2, Up and about >50% of waking hours).

**Table 2:** Hazard ratio by Cox regression analysis.

		OS			PFS		
		HR	95% CI	*p*-Value	HR	95% CI	*p*-Value
Response	CR or PR	1.000			1.000		
	SD or PD	3.918	1.955–7.849	< 0.001	4.478	2.206–9.089	< 0.001
APOBEC3B	Low	1.000			1.000		
	High	0.292	0.118–0.723	0.008	0.335	0.139–0.806	0.015

**Table 3:** Association between APOBEC3B expression and CD8+/CD3+ ratio.

Intra-Tumoral and Stromal TIL Number				
CD8/CD3 ratio	Total	APOEC3B low	APOBEC3B high	*p* value
Low	52	17	35	0.039
High	42	6	36	
**Intra-Tumoral and Stromal TIL Area**				
CD8/CD3 ratio	Total	APOBEC3B low	APOBEC3B high	*p* value
Low	52	17	35	0.039
High	42	6	36	

**Table 4:** Chemotherapy response according to APOBEC3B expression.

	Total	APOBEC3B * Low	APOBEC3B ** High	*p*-Value
CR or PR	59	12	24	0.227
SD or PD	35	11	47	
CR, PR or SD	73	15	58	0.099
PD	21	8	13	
	Total	APOBEC3B low	APOBEC3B high	
CR	18	2	16	
PR	41	10	31	
SD	14	3	11	
PD	21	8	13	
Total	94	23	71	

* Low: H-score ≤ 90, ** High, H-score > 90, H-score (nucleus + cytoplasm) = intensity × proportion. CR, complete response; PR, partial response; SD, stable disease; PD, progressive disease, these terms of response evaluation follow the definition of RECIST criteria v1.1.

## Data Availability

Data sharing is not applicable to this article due to ethical restrictions.

## Supplementary Materials

The following are available online at https://www.mdpi.com/article/10.3390/curroncol28030154/s1, Figure S1: Survival curves according to TILs, Table S1: Univariate analysis of survival by log-rank test, Table S2: The association between APOBEC3B expression and FOXP3+/CD4+ TIL ratio, Table S3: chemotherapy response according to TILs.

## References

[1] Reuter V.E. (2006). The pathology of bladder cancer. Urology.

[2] Antoni S., Ferlay J., Soerjomataram I., Znaor A., Jemal A., Bray F. (2017). Bladder Cancer Incidence and Mortality: A Global Overview and Recent Trends. Eur. Urol..

[3] von der Maase H., Sengelov L., Roberts J.T., Ricci S., Dogliotti L., Oliver T., Moore M.J., Zimmermann A., Arning M. (2005). Long-term survival results of a randomized trial comparing gemcitabine plus cisplatin, with methotrexate, vinblastine, doxorubicin, plus cisplatin in patients with bladder cancer. J. Clin. Oncol..

[4] Bellmunt J., Powles T., Vogelzang N.J. (2017). A review on the evolution of PD-1/PD-L1 immunotherapy for bladder cancer: The future is now. Cancer Treat. Rev..

[5] Dogliotti L., Carteni G., Siena S., Bertetto O., Martoni A., Bono A., Amadori D., Onat H., Marini L. (2007). Gemcitabine plus cisplatin versus gemcitabine plus carboplatin as first-line chemotherapy in advanced transitional cell carcinoma of the urothelium: Results of a randomized phase 2 trial. Eur. Urol..

[6] Cumberbatch M.G.K., Noon A.P. (2019). Epidemiology, aetiology and screening of bladder cancer. Transl. Androl. Urol..

[7] Lawrence M.S., Stojanov P., Polak P., Kryukov G.V., Cibulskis K., Sivachenko A., Carter S.L., Stewart C., Mermel C.H., Roberts S.A. (2013). Mutational heterogeneity in cancer and the search for new cancer-associated genes. Nature.

[8] Weinstein J.N., Lerner S.P., Kwiatkowski D.J. (2014). Comprehensive molecular characterization of urothelial bladder carcinoma. Nature.

[9] Tate J.G., Bamford S., Jubb H.C., Sondka Z., Beare D.M., Bindal N., Boutselakis H., Cole C.G., Creatore C., Dawson E. (2019). COSMIC: The Catalogue of Somatic Mutations in Cancer. Nucleic Acids Res..

[10] Vlachostergios P.J., Faltas B.M. (2018). Treatment resistance in urothelial carcinoma: An evolutionary perspective. Nat. Rev. Clin. Oncol..

[11] Roberts S.A., Lawrence M.S., Klimczak L.J., Grimm S.A., Fargo D., Stojanov P., Kiezun A., Kryukov G.V., Carter S.L., Saksena G. (2013). An APOBEC cytidine deaminase mutagenesis pattern is widespread in human cancers. Nat. Genet..

[12] Glaser A.P., Fantini D., Wang Y., Yu Y., Rimar K.J., Podojil J.R., Miller S.D., Meeks J.J. (2018). APOBEC-mediated mutagenesis in urothelial carcinoma is associated with improved survival, mutations in DNA damage response genes, and immune response. Oncotarget.

[13] Baras A.S., Drake C., Liu J.J., Gandhi N., Kates M., Hoque M.O., Meeker A., Hahn N., Taube J.M., Schoenberg M.P. (2016). The ratio of CD8 to Treg tumor-infiltrating lymphocytes is associated with response to cisplatin-based neoadjuvant chemotherapy in patients with muscle invasive urothelial carcinoma of the bladder. Oncoimmunology.

[14] Robertson A.G., Kim J., Al-Ahmadie H., Bellmunt J., Guo G., Cherniack A.D., Hinoue T., Laird P.W., Hoadley K.A., Akbani R. (2017). Comprehensive Molecular Characterization of Muscle-Invasive Bladder Cancer. Cell.

[15] Mullane S.A., Werner L., Rosenberg J., Signoretti S., Callea M., Choueiri T.K., Freeman G.J., Bellmunt J. (2016). Correlation of Apobec Mrna Expression with overall Survival and pd-l1 Expression in Urothelial Carcinoma. Sci. Rep..

[16] Kanu N., Cerone M.A., Goh G., Zalmas L.P., Bartkova J., Dietzen M., McGranahan N., Rogers R., Law E.K., Gromova I. (2016). DNA replication stress mediates APOBEC3 family mutagenesis in breast cancer. Genome. Biol..

[17] Zhang J., Dai Q., Park D., Deng X. (2016). Targeting DNA Replication Stress for Cancer Therapy. Genes.

[18] Buisson R., Lawrence M.S., Benes C.H., Zou L. (2017). APOBEC3A and APOBEC3B Activities Render Cancer Cells Susceptible to ATR Inhibition. Cancer Res..

[19] Jiang T., Shi J., Dong Z., Hou L., Zhao C., Li X., Mao B., Zhu W., Guo X., Zhang H. (2019). Genomic landscape and its correlations with tumor mutational burden, PD-L1 expression, and immune cells infiltration in Chinese lung squamous cell carcinoma. J. Hematol. Oncol..

[20] Faraj S.F., Munari E., Guner G., Taube J., Anders R., Hicks J., Meeker A., Schoenberg M., Bivalacqua T., Drake C. (2015). Assessment of tumoral PD-L1 expression and intratumoral CD8+ T cells in urothelial carcinoma. Urology.

[21] Denkert C., Loibl S., Noske A., Roller M., Muller B.M., Komor M., Budczies J., Darb-Esfahani S., Kronenwett R., Hanusch C. (2010). Tumor-associated lymphocytes as an independent predictor of response to neoadjuvant chemotherapy in breast cancer. J. Clin. Oncol..

[22] Leonard B., Starrett G.J., Maurer M.J., Oberg A.L., Van Bockstal M., Van Dorpe J., De Wever O., Helleman J., Sieuwerts A.M., Berns E.M. (2016). APOBEC3G Expression Correlates with T-Cell Infiltration and Improved Clinical Outcomes in High-grade Serous Ovarian Carcinoma. Clin. Cancer Res..

[23] Kroemer G., Galluzzi L., Kepp O., Zitvogel L. (2013). Immunogenic cell death in cancer therapy. Annu. Rev. Immunol..

[24] Eisenhauer E.A., Therasse P., Bogaerts J., Schwartz L.H., Sargent D., Ford R., Dancey J., Arbuck S., Gwyther S., Mooney M. (2009). New response evaluation criteria in solid tumours: Revised RECIST guideline (version 1.1). Eur. J. Cancer.

[25] Xu L., Chang Y., An H., Zhu Y., Yang Y., Xu J. (2015). High APOBEC3B expression is a predictor of recurrence in patients with low-risk clear cell renal cell carcinoma. Urol. Oncol..

[26] Huang H.S., Su H.Y., Li P.H., Chiang P.H., Huang C.H., Chen C.H., Hsieh M.C. (2018). Prognostic impact of tumor infiltrating lymphocytes on patients with metastatic urothelial carcinoma receiving platinum based chemotherapy. Sci. Rep..

[27] Ishibashi H., Suzuki T., Suzuki S., Moriya T., Kaneko C., Takizawa T., Sunamori M., Handa M., Kondo T., Sasano H. (2003). Sex steroid hormone receptors in human thymoma. J. Clin. Endocrinol. Metab..

