# Supplementary material for: Prognostic Impact of APOBEC3B Expression in Metastatic Urothelial Carcinoma and Its Association with Tumor-Infiltrating Cytotoxic T Cells
Source: Curr Oncol. 2021 Apr 28;28(3):1652–62. doi: 10.3390/curroncol28030154 (PMC8161743; doi:10.3390/curroncol28030154)
Supplement: Supplementary file 1 [file curroncol-28-00154-s001.zip › curroncol-1129749-supplementary.pdf]

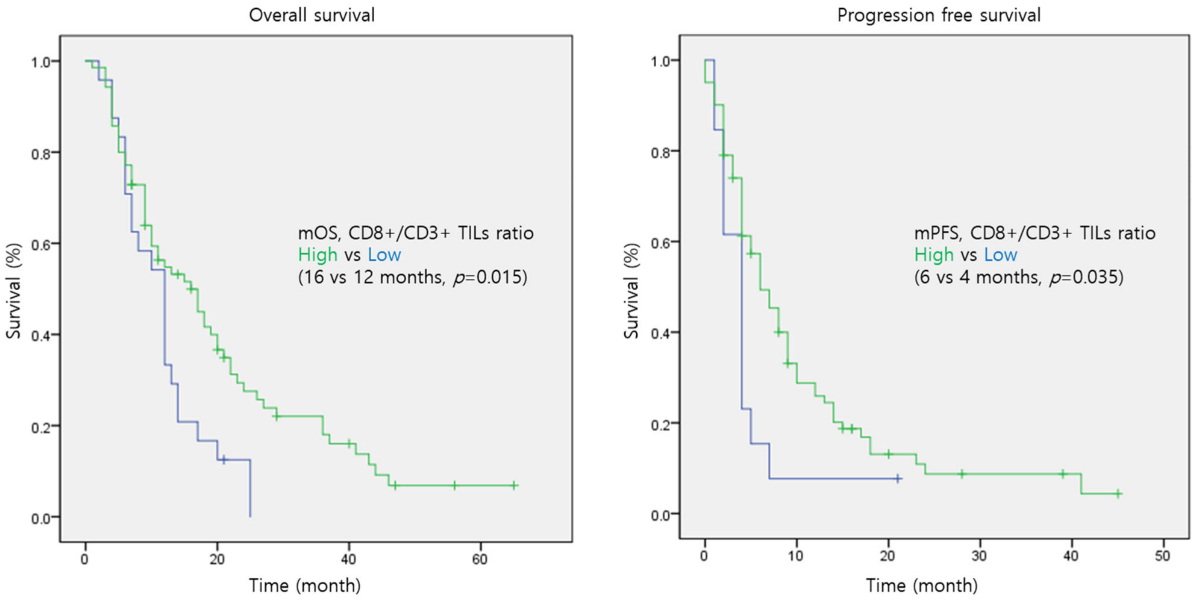

Figure S1. Survival curves according to TILs.

Table S1. Univariate analysis of survival by log-rank test.

		mOS (months)	*p* value	mPFS (months)	*p* value
ECOG	0	23	0.001	8	0.026
	1	14		7	
	2	7		4	
Chemotherapy response	CR or PR	18	<0.001	9	<0.001
	SD or PD	6		2	
Subsequent chemotherapy	No	10	0.767	6	0.024
	Yes	15		6	
Surgery	No	10	0.004	4	0.011
	Yes	17		7	

Table S2. The association between APOBEC3B expression and FOXP3+/CD4+ TIL ratio.

**Intratumoral and stromal TILs number**				
FOXP3/CD4 ratio	Total	A3B low	A3B high	*p* value
Low	71	15	56	0.186
High	23	8	15	
**Intratumoral and stromal TILs area**				
FOXP3/CD4 ratio	Total	A3B low	A3B high	*p* value
Low	71	15	56	0.186
High	23	8	15	

Table S3. Chemotherapy response according to TILs.

	**Total**	**TILs* low**	**TILs high**	***p* value**
CR or PR	59	36	23	0.149
SD or PD	35	16	19	
CR, PR or SD	73	41	32	0.759
PD	21	11	10	
	**total**	**TILs low**	**TILs high**	***p* value**
CR or PR	59	34	25	0.559
SD or PD	35	18	17	
CR, PR or SD	73	41	32	0.759
PD	21	11	10	

*TILs: intratumoral + stromal CD8+/CD3+ TILs ratio.
